# Editorial: Rising stars in: consciousness research 2021

**DOI:** 10.3389/fpsyg.2023.1205982

**Published:** 2023-05-16

**Authors:** Xerxes D. Arsiwalla, Narayanan Srinivasan, Luca Simione, Johannes Kleiner, Antonino Raffone

**Affiliations:** ^1^Department of Information and Communication Technologies, Pompeu Fabra University, Barcelona, Spain; ^2^Association for Mathematical Consciousness Science, Munich, Germany; ^3^Department of Cognitive Science, Indian Institute of Technology Kanpur, Kanpur, India; ^4^Institute of Cognitive Sciences and Technologies, Consiglio Nazionale delle Ricerche, Rome, Italy; ^5^Faculty of Interpreting and Translation, UNINT Università degli Studi Internazionali di Roma, Rome, Italy; ^6^Munich Center for Mathematical Philosophy, Ludwig Maximilian University of Munich, Munich, Germany; ^7^Munich Graduate School of Systemic Neurosciences, Ludwig Maximilian University of Munich, Munich, Germany; ^8^Department of Psychology, Sapienza University of Rome, Rome, Italy

**Keywords:** contents of consciousness, sense of self, embodied cognition, hypnosis, meditation, criticality, higher-order theories, monism

The Rising Stars topic serves as a forum for new directions and emerging trends in consciousness research, showcasing high-quality work of early-stage researchers. Compared to platforms highlighting dominant paradigms and established viewpoints (Srinivasan et al., 2023), this Research Topic presents new independent ideas with promising scientific impact. It highlights the diversity of research performed across the entire breadth of consciousness science, including advances in theory, experiment, methodology and clinical practice.

The article by Oblak et al. report an exploratory phenomenological study on the experience of individuals during task performance, as opposed to purely behavioral assessments (reaction-times/accuracy). The authors used a working memory visuo-spatial change detection task to explore experiences elicited by and accompanying task performance. Implementing contemporary phenomenological and qualitative methodology, they gathered subjective reports during the memory task. Qualitative reports revealed rich experiential landscapes associated with task-performance, suggesting a distinction between two broad classes of experience: phenomena at the front of consciousness and background feelings. The former captured cognitive strategies and aspects of metacognition, whereas the latter encapsulated difficult-to-detect aspects of experience that comprise the overall sense of experience (bodily feelings, emotional atmosphere, mood). This study focused primarily on background feelings of subjects and the nature of the elicited experience.

Bréchet's article looks at how personal memories and bodily-cues influence our sense of self. Bréchet explores the underlying characteristics of self-consciousness and its relation to bodily signals and episodic memory. This article outlines recent behavioral and neuroimaging evidence indicating that bodily cues play a fundamental role in autobiographical memory. The author also discusses emerging concepts regarding the current understanding of bodily-self and autobiographical-self, and their links to self-consciousness. These ideas can further help bridge theoretical notions of self (Metzinger, 2004) to empirical underpinnings.

The article by Dapor et al. investigates bodily states in relation to consciousness. They reviewed the boundaries between the physical and social world, and shed insights on the intersection between those. This review focused on interactions between sensory inputs and social cognition in visual perception mediated by body indices, such as gait and posture. For instance, depending on our arm's length, height and capacity of movement, we create our own image of the world based on a continuous compromise between sensory inputs and expected behavior. The authors propose that we use our bodies as natural “rulers” to measure the physical and social world around us. This goes beyond approaches that define perception as stimulus-centered, and emphasizes an embodied agent-based perspective, which closely resonates with paradigms of enactivism and predictive coding (Clark, 2013; Varela et al., 2016).

Toward improving the quality of life of post-treatment cancer patients, Grégoire et al. presented a study protocol for administering complementary therapeutic approaches in addition to standard cancer therapies. These included hypnosis, meditation and self-induced cognitive trance. The authors detail the protocol of a preference-based longitudinal controlled trial that assessed the effectiveness of the stated interventions on the common symptom cluster associated with oncological patients: cancer-related fatigue, emotional distress, sleep, pain, and cognitive difficulties using questionnaires and neurobiological measures.

Schmidt's article shows how hypnosis elicits positive feelings of safety for people undergoing substance abuse therapy or those in highly stressful environments, such as intensive care units. Patients tolerated non-invasive ventilation much better when they got the suggestion to feel safe. The effects of positive therapeutic suggestions delivered during hypnosis even persist afterwards. Post-hypnotic suggestions are associations between a certain emotional state and a trigger that elicits this emotional state after hypnosis is over. Post-hypnotic suggestions of safety were shown to be effective weeks after therapy. Such studies underscore the potential of manipulating non-standard states of consciousness for realizing new clinical therapies applicable to a broad range of diseases.

The article by Rabuffo et al. propose spontaneous neuronal avalanches as correlates of access consciousness. This draws upon the idea that the brain self-regulates around a critical point, at the edge of a phase transition (Cocchi et al., 2017). This has also been linked to optimal information processing and consciousness (Werner, 2009; Shew et al., 2011; Arsiwalla and Verschure, 2016; Arsiwalla et al., 2017). Based on the observation that spontaneous brain activity dynamically switches between epochs of segregation and large-scale integration of information, the authors hypothesize a brain-state dependence of conscious access, whereby the presence of either segregated or integrated states marks distinct modes of information processing. They propose a test experiment to validate the hypothesis that conscious access occurs in aperiodic cycles; namely, alternating windows where new incoming information is collected but not experienced, to punctuated short-lived integration events, where conscious access to previously collected content occurs. In particular, they suggest that integration events correspond to neuronal avalanches, which are collective bursts of neuronal activity ubiquitously observed in electrophysiological recordings. If confirmed, this framework could link the physics of spontaneous cortical dynamics to the concept of ignition (Mashour et al., 2020) within the global neuronal workspace theory, whereby conscious access correlates to bursts of neuronal activity.

Kirkeby-Hinrup's article discusses the plausibility of misrepresentation of conscious states in the context of higher-order theories. The argument relies on the assumption that conscious states are generated by processes in the brain. The underlying idea is that if the brain generates conscious states then misrepresentations can occur. The reason for this is that brain states can be corrupted and, accordingly, a conscious state that is at least partly caused by a corrupted brain state may be a misrepresentation. Furthermore, given that corruption of neural states is both possible and relatively frequent, the author argues that it is plausible that occasionally such corruption may result in misrepresentation.

The article by Lahav and Neemeh proposes that phenomenal consciousness manifests as a relativistic, rather than an absolute property. These authors contend that both the dualist and illusionist positions are flawed because those tacitly assume consciousness to be an absolute property that is observer-independent. This work proposes a conceptual and a mathematical argument for a relativistic theory of consciousness in which a system either has or doesn't have phenomenal consciousness with respect to some observer. The authors argue that in the frame of reference of the cognitive system, phenomenal properties will be observable (first-person perspective) and in other frames of reference it will not (third-person perspective). These two cognitive frames of reference are both deemed correct, just as the case of an observer claiming to be stationary when another would conclude that the former has constant velocity. Assuming consciousness is a relativistic phenomenon, neither observer's position is privileged. They both describe the same reality. This partially relates to reflexive monism (Velmans, 2009). The authors then seek to bridge the explanatory gap and address the hard problem.

In summary, this Research Topic has brought together diverse works addressing important issues in consciousness research such as the phenomenology of experience, the empirical underpinnings of the self, the role of sociality, the use of non-standard states of consciousness for designing new clinical therapies, a possible mechanism bridging cortical dynamics to access consciousness, an argument on misrepresentation in the context of higher-order theories, and a new hypothesis advocating observer-dependence of phenomenological properties of consciousness. Given the broad scope and rapid growth of the science of consciousness, it is imperative that independent perspectives different from conventional paradigms, are provided with a suitable platform for dissemination. This Research Topic addresses that need.

## Author contributions

All authors listed have made a substantial, direct, and intellectual contribution to the work and approved it for publication.
